# Steroid-Induced Ocular Hypertension in Mice Is Differentially Reduced by Selective EP2, EP3, EP4, and IP Prostanoid Receptor Agonists

**DOI:** 10.3390/ijms25063328

**Published:** 2024-03-15

**Authors:** Najam A. Sharif, J. Cameron Millar, Gulab Zode, Takashi Ota

**Affiliations:** 1 Ophthalmology Innovation Center, Santen Inc., Emeryville, CA 94608, USA; nsharif@nanostherapeutics.com; 2 Institute of Ophthalmology, University College London (UCL), London EC1V 9EL, UK; 3 Department of Ophthalmology, Imperial College of Science and Technology, St. Mary’s Campus, London W2 1PG, UK; 4 Department of Ophthalmology, Eye-ACP Duke-National University of Singapore Medical School, Singapore 119228, Singapore; 5 Singapore Eye Research Institute (SERI), Singapore 168751, Singapore; 6 Department of Pharmacy Sciences, Creighton University, Omaha, NE 68178, USA; 7 Department of Pharmaceutical Sciences, College of Pharmacy and Health Sciences, Texas Southern University, Houston, TX 77004, USA; 8 Department of Pharmacology & Neuroscience, North Texas Eye Research Institute (NTERI), University of North Texas Health Science Center, Fort Worth, TX 76107, USA; 9 Department of Ophthalmology and Center for Translational Vision Research, University of California, Irvine, CA 92617, USA; gzode@hs.uci.edu; 10 Ophthalmology Innovation Center, Santen Pharmaceutical Co., Ltd., Nara 630-0101, Japan

**Keywords:** steroid, IOP, ocular hypertension, EP2 receptor agonist, Butaprost, PF-04217329

## Abstract

We tested five chemically and metabolically stable prostaglandin (PG) receptor agonists in a mouse model of dexamethasone-induced ocular hypertension (OHT). Whilst all compounds significantly (*p* < 0.05, ANOVA) lowered intraocular pressure (IOP) after twice-daily bilateral topical ocular dosing (5 µg/dose) over three weeks, the time course and magnitude of the responses varied. The onset of action of NS-304 (IP-PG receptor agonist) and rivenprost (EP4-PG receptor agonist) was slower than that of misoprostol (mixed EP2/EP3/EP4-PG receptor agonist), PF-04217329 (EP2-PG receptor agonist), and butaprost (EP2-PG receptor agonist). The rank order of IOP-lowering efficacies aligned with the onset of actions of these compounds. Peak IOP reductions relative to vehicle controls were as follows: misoprostol (74.52%) = PF-04217329 (74.32%) > butaprost (65.2%) > rivenprost (58.4%) > NS-304 (55.3%). A literature survey indicated that few previously evaluated compounds (e.g., latanoprost, timolol, pilocarpine, brimonidine, dorzolamide, cromakalim analog (CKLP1), losartan, tissue plasminogen activator, trans-resveratrol, sodium 4-phenyl acetic acid, etc.) in various animal models of steroid-induced OHT were able to match the effectiveness of misoprostol, PF-04217329 or butaprost. Since a common feature of the latter compounds is their relatively high affinity and potency at the EP2-PG receptor sub-type, which activates the production of intracellular cAMP in target cells, our studies suggest that drugs selective for the EP2-PG receptor may be suited to treat corticosteroid-induced OHT.

## 1. Introduction

The degenerative processes that cause optic nerve and retinal dysfunction leading to serious visual impairment, and which can lead to irreversible blindness if not timely slowed or prevented, are the result of a collection of multifactorial chronic ocular diseases called “glaucoma” [1,2,3,4]. Two major types of glaucoma exist (primary and secondary), under which are a subset of other glaucomatous optic neuropathies (GON). Primary glaucoma encompasses open-angle glaucoma (OAG) and angle-closure glaucoma (ACG) types, which are designated based on whether there is space between the iris and the cornea at the angle of the anterior chamber (ANC) through which the aqueous humor (AQH) can pass in order to be drained from the ANC. When the corneo-iridial space is reduced due to the collapse of the iris or inflammatory bulging of the iris (iritis), the angle of the ANC is either highly restricted or closed [1,2,3]. In the open-angle case, if the AQH drainage system (trabecular meshwork [TM]) becomes clogged due to the aging process and/or due to pathological accumulation of collagen, fibronectin and other extracellular matrix (ECM) components, the resistance to AQH efflux from the ANC gradually rises, and thus also the intraocular (fluid) pressure (IOP) [5], thus leading to ocular hypertension (OHT). About 80% of primary glaucomas are in the OAG category.

The detrimental effects of OHT are felt particularly at the structurally weak optic nerve head (ONH) and lamina cribrosa (LC) at the retinal/optic nerve levels [1,2,3,6]. Many inflammatory cytokines, endothelin, glutamate, and matrix metalloproteinases released at the latter tissues due to the compressive and stretching effects of the elevated IOP conspire to damage the retinal ganglion cell (RGC) axons (which form the neural connection between the eye and the brain), and their cell bodies over time [7,8,9]. These deleterious events result in a progressive diminution of color and contrast sensitivity and blurry and patchy loss of visual field in the affected eye(s) of the patient, and if untreated, eventually lead to tunnel vision and, later, blindness [1,6,7,8,9]. Since the disease is painless and asymptomatic until substantial loss of RGCs and optic nerve damage has occurred, the patient can lose a significant degree of vision if IOP-lowering treatment is not initiated at the start of visual field deterioration [10]. Preservation of the remaining retina–optic nerve–visual brain center connectivity is now of utmost importance so that further damage to the RGCs is reduced or halted [4,6,11]. The immediate treatment upon diagnosis of OAG is to have the patient instill daily eyedrops of ocular hypotensive drugs and reduce the IOP since much pre-clinical and clinical experience has directly linked abnormally elevated IOP to vision loss [1,3,12,13,14,15,16,17].

**Figure 1:**
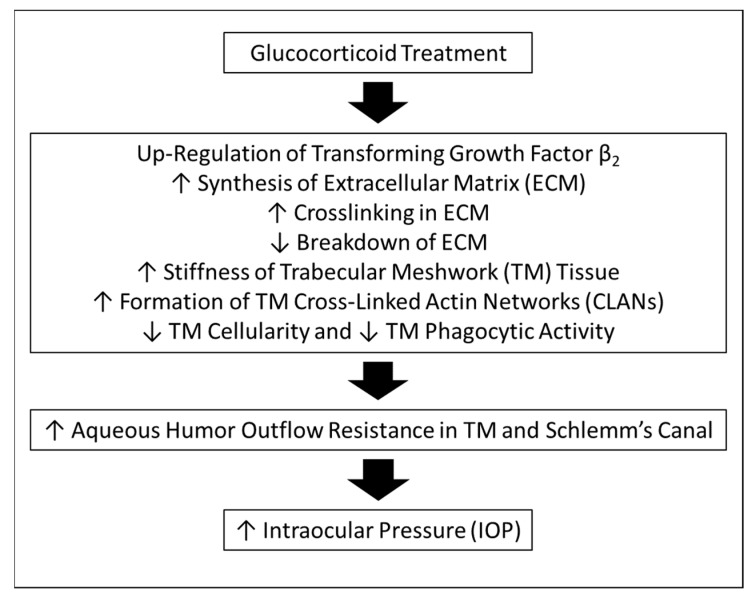
Schematic of multiple mechanisms of glucocorticoid-induced elevation of intraocular pressure (IOP). Vertical arrows indicate the following: ↑ (Increased); ↓ (Decreased).

Glucocorticoids are powerful drugs that are used to treat a variety of inflammatory and immunological diseases of the body. In the eye, glucocorticoids such as dexamethasone (DEX), prednisolone, and triamcinolone are effective against a variety of inflammatory conditions [18]. Unfortunately, the use of these compounds can lead to side effects that adversely affect the eye, including cataract formation and, in steroid responders, the elevation of IOP [18,19] (Figure 1). Indeed, in a retrospective clinical study comparing the effects of various steroids dosed intravitreally (ivt) and/or via sub-tenons (s.t.), these drugs significantly increased IOP in human subjects. In 31% of patients with persistently raised IOP, topical ocular hypotensive drug therapy had to be continued over the almost 2-year follow-up period [18].

The major negative impact of the steroids occurs in the TM/Schlemm’s canal area of the conventional AQH outflow pathway [20,21,22,23,24,25]. Thus, the TM becomes congested due to pathological up-regulation of transforming growth factor beta-1/2, which induces accumulation of ECM components [26], with further increases in outflow resistance due to deposition of glycosaminoglycans, elastin, and cross-linking of proteins (including cross-linked actin networks (CLANS)), which all reduce AQH outflow facility and raise the IOP [21]. The concomitant reduction in TM flexibility [27] and cell phagocytic activity [28] and decreases in local tissue plasminogen activator, stromelysin, and the activity of several TM metalloproteases further exacerbate the OHT problem [28,29]. Prolonged steroid treatments can lead to IOP-dependent glaucomatous retinal neurodegeneration, including significant loss of RGCs and their axons and, thus, vision loss [20].

Current treatments for steroid-induced OHT rely on existing topical ocular hypotensive drugs. The first-line therapeutic agent is the FP receptor agonist latanoprost, although beta-blockers, alpha-2 agonists, topically active carbonic anhydrase inhibitors (CAIs), miotics, and rho-kinase inhibitors may also be used [30]. However, all these drugs have many side effects that limit their clinical utility, including burning and stinging of the ocular surface, hyperemia, allergic conjunctivitis, cystoid macular edema pulmonary insufficiency, bradycardia, tinnitus, gastrointestinal disturbances, bad taste, depression, anxiety, etc. [31,32,33]. Additionally, ≥30% of patients do not respond well to drug therapy, especially FP receptor agonists [1,16,17]. Uncontrolled elevated IOP and IOP spikes may even require laser trabeculoplasty, surgical intervention, and/or microshunt device implantation to relieve the OHT [34,35]. Consequently, there is a critical need to find more effective drugs with reduced side-effect profiles and improved therapeutic indices to treat glucocorticoid-induced OHT.

**Figure 2:**
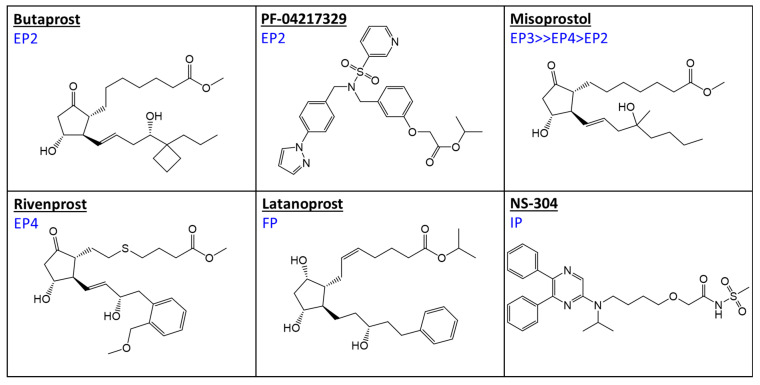
Chemical structures of the major prostaglandin receptor type agonists used and/or discussed in this article. The blue labels indicate the preference of the compound to primarily interact with the particular prostanoid receptor type or sub-type shown over the other receptors in this family.

A recent survey of literature spanning >2 decades revealed that EP2-prostaglandin receptor agonists have a rapid mode of action in terms of IOP-lowering in many animal models of OHT/glaucoma [16]. Based on those findings, we now wanted to explore whether a range of PGs (Figure 2) that have relative selectivity for and which activate EP2, EP3, EP4, and IP receptors (Table 1) may be suitable for lowering IOP in a steroid-induced mouse model of OHT. In order to ensure adequate penetration of the topical ocularly delivered compounds, the pro-drug molecules of each compound (esters) were utilized in the current studies.

Many animal models have been developed, characterized, and utilized to study the pathogenic elements of OHT and glaucoma [39,40,41,42,43,44,45,46,47,48,49,50,51,52,53,54,55] (Table 2). We chose to use the mouse in the present study. Use of the mouse is justified on the grounds that it possesses an ocular anterior segment that is anatomically and physiologically very similar to that of the human, more so than any laboratory animal species except non-human primates (NHPs) and rats. In addition, the arrangement of blood vessels, innervation, pharmacologic receptor expression profile, and the conventional outflow pathway all closely mirror that of the human. The conventional aqueous humor outflow pathway includes a three-layered trabecular meshwork, incorporating uveal, corneoscleral, and juxtacanalicular regions, which is very similar to the human arrangement, unlike in many mammalian species, which have a trabecular meshwork which does not appear to be arranged as three separate histologic layers. A true circular Schlemm’s canal is also present, along with scleral collector channels and anterior aqueous veins, once again, also unlike the situation in most mammalian species, which instead have a discontinuous anterior aqueous venous plexus. C57BL/6J mice respond to weekly periocular injections of DEX with changes in the trabecular meshwork and elevation of intraocular pressure, very similar to those seen in human steroid responders. In addition, there is much consensus within the ocular community that the mouse model of steroid-induced glaucoma most closely resembles the human form of steroid-induced glaucoma [42]. Additionally, mice are easier to handle than other large animals.

Indeed, there have been at least seven sets of recent studies where mice (mostly C57BL/6J) have been used to induce OHT using DEX either delivered by injection via subconjunctival injection [56,57], periocular injection at the conjunctival fornix [20], topical ocular [24,58,59,60] or via mini-osmotic pumps [61,62,63] routes of administration. Additionally, a variety of classes of pharmacological agents and plant-derived extracts have shown IOP-lowering efficacy in the mouse models of steroid-induced OHT, thereby lending further credibility to this particular model (Table 2). Therefore, we used the recently established and validated mouse model of periocular-delivered DEX [20] to evaluate the IOP-reducing actions of the five receptor-selective prostaglandins over several weeks of topical ocular treatment. However, unlike in the latter report, in which the mice were anesthetized for IOP readings [20], in the present studies, the animals were conscious throughout all IOP measurements. In the current studies, the dose of the compounds was chosen based on historic dose–response data obtained from various models of OHT and or naïve animals (e.g., Table 2; Refs. [16,20,30,37,38,61,64,65,66,67,68,69,70,71,72,73,74,75]) and the relative selectivity of the compounds for their cognate, most preferred receptor types or sub-types (Table 3). Ester pro-drugs were also selected to ensure suitable topical ocular penetration of the drug and release of the active moiety into the AQH.

**Table 2:** Literature data on compounds or treatment modalities that lower IOP in steroid-induced OHT in various animal models, human subjects, and data obtained from the current studies for comparison.

Animal Model or Human Subjects	Route of Administration of Steroid	Steroid Used	Test Compound	Max. IOP Reduction	Reference
C57BL/6 mice	Topical ocular	Dexamethasone	Sodium 4-phenylacetate (Chaperone)	16%	[24]
C57BL/6J mice	Periocular fornix	Dexamethasone	Cosopt + latanoprost (CAI inhibitor + Timolol + FP–PGR agonist)	187%	[20]
C57BL/6J mice	Topical ocular		Metformin (anti-diabetic/glycemic)	14%	[59]
C57BL/6J mice	Topical ocular	Dexamethasone	HL-3501 (Adenosine-3R antagonist) Latanoprost (FP–PGR agonist)	37% 36%	[58]
C57BL/6J mice	Subconjunctival (Fornix)	Dexamethasone	CKLP1 (Cromakalim analog (K+-channel activator)) Diazoxide (K+-channel activator; anti-glycemic)	24% 32%	[56]
C57BL/6J mice	Subconjunctival (Fornix)	Dexamethasone	Stanniocalcin-1 (hormone)	26%	[57]
C57BL/6J mice	Mini-osmotic pumps (systemic)	Dexamethasone	siRNA to zonula occluddens-1 and tricellulin	~20%	[63]
C57BL/6J mice	Topical ocular	Dexamethasone	Netarsudil (Rho kinase inhibitor)	20%	[60]
C57BL/6J mice	Mini-osmotic pumps	Dexamethasone	Cabergoline (D3 and 5HT2 receptor agonist)	40%	[62]
129.B6 mice	Mini-osmotic pumps	Dexamethasone	Timolol (β-blocker) Latanoprost (FP–PGR agonist) Y39983 (Rho kinase inhibitor)	16% 27% 33%	[61]
**C57BL/6J mice**	**Subconjunctival (Fornix)**	**Dexamethasone**	**Misoprostol (EP3-EP4-EP2R agonist)** **PF-04217329 (EP2 R agonist)** **Butaprost (EP2R agonist)** **Rivenprost (EP4R agonist)** **NS-304 (IPR-agonist)**	**74.5%** **74.3%** **65.2%** **58.4%** **55.3%**	**The current studies**
Sprague-Dawley Rats	Topical ocular	Dexamethasone	Timolol (βR-blocker) Pilocarpine (Muscarinic R agonist) Dorzolamide (CAI) Brimonidine (α2-R agonist) Latanoprost (FP–PGR agonist)	20% 16% 19% 17% 27%	[45]
Sprague-Dawley Rats	Topical ocular	Dexamethasone	Losartan (Ang. IIR antagonist)	27%	[47]
Sprague-Dawley Rats	Subconjunctival (Fornix)	Betamethasone	C. cicadae mycelia extract	50%	[48]
Rabbits	Topical ocular	Prednisolone	Ocimum bascilicum seed extract	32%	[51]
Rabbits	Topical ocular	Prednisolone	Aegle Marmelos fruit extract	28%	[50]
Rabbits	Topical ocular	Prednisolone	Pilocarpine (Muscarinic R agonist) Timolol (βR-blocker) Latanoprost (FP–PGR agonist)	26% 34% 35%	[49]
Sheep	Topical ocular	Prednisolone	Tissue plasminogen activator (tPA) (intracameral)	36%	[53]
Sheep	Sub-tenon	Triamcinolone	MMP-1 gene therapy	70%	[52]
Sheep	Topical ocular or Intravitreal	Prednisolone or Difluprednate Triamcinolone	MMP-1 gene therapy	38%	[54]
Human	Topical/systemic	Dexamethasone	Latanoprost (FP–PGR agonist)	28%	[30]

Data from the literature and our study data are presented for comparison purposes. CAI, carbonic anhydrase inhibitor; PG, prostaglandin; R, receptor.

## 2. Results

**Figure 3:**
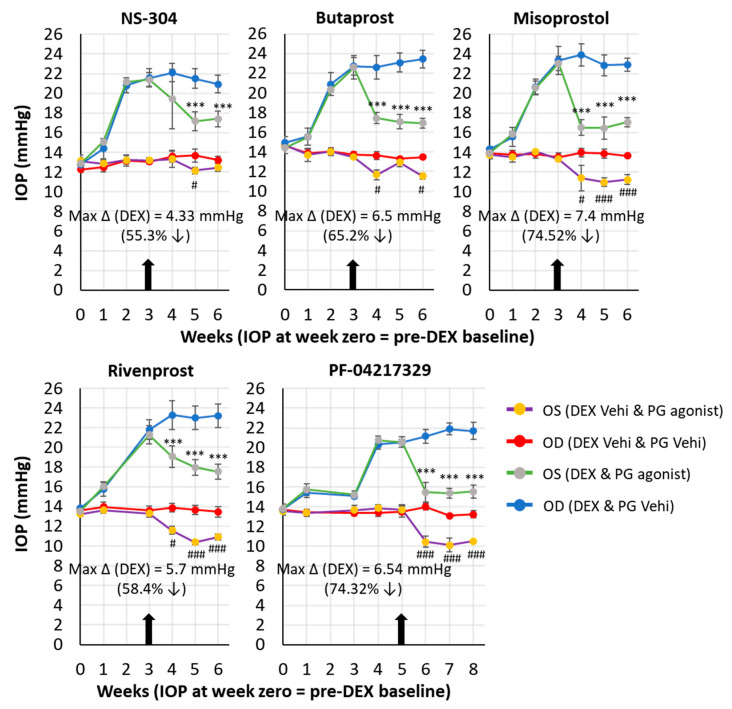
The ability of various prostaglandin receptor-type-selective agonist compounds to reduce IOP in DEX-induced OHT in mice is shown. The vertical arrow indicates when twice-daily topical ocular dosing commenced, which then continued for the remainder of the experiment. Data are mean ± SDM from 5 mice/group. In all cases, weekly periocular injections of DEX significantly elevated IOP (*p* < 0.001). Also, in all cases, subsequent drug treatment significantly reduced IOP in both the DEX OHT eyes and the ocular normotensive control eyes. *** *p* < 0.001; the significance of difference between PG agonist and corresponding PG Vehi groups in DEX-injected eyes; ^#^ *p* < 0.05; ^###^ *p* < 0.001; the significance of difference between PG agonist and corresponding PG Vehi groups in DEX Vehi injected eyes, as indicated by 2-Factor ANOVA followed by the Holm-Sidak post-hoc test. Vertical arrow (↓) indicates maximum % reduction in IOP in DEX group following topical ocular drug treatment.

In one cohort of five animals, once-weekly injections of DEX-Veh took place in both eyes (OU). IOP was not elevated in these animals. After the 4th DEX-Veh injection (or 5th, in the case of PF-04217329), the topical ocular drug was applied to the left eye (OS) on a twice-daily basis, while the topical ocular vehicle was applied to the right eye (OD). This proceeded for the remainder of the experiment. The red line shows the IOP response to the topical ocular vehicle (twice daily) in the DEX-Veh injected eyes (Figure 3). The purple line shows the response to topical ocular drug (twice daily) in the DEX-Veh injected eyes (Figure 3). In a separate cohort of an additional five animals, once-weekly injections of DEX were conducted in both eyes (OU). IOP was elevated in these animals. After the 4th DEX injection (or 5th, in the case of PF-04217329), the topical ocular drug was applied to the left eye (OS) on a twice-daily basis, while the topical ocular vehicle was applied to the right eye (OD). This proceeded for the remainder of the experiment. The light blue line shows the IOP response to topical ocular Vehicle (BID) in the DEX-injected eyes. The light green line shows the response to topical ocular drug (BID) in the DEX-injected eyes.

Cohorts of mice receiving periocular injections of dexamethasone acetate (DEX; 10 mg/mL) (20 μL; 200 μg) once-weekly in both eyes exhibited elevations of IOP, which peaked around 4–5 weeks after treatment and essentially plateaued over the next 3–4 weeks. Starting with baseline IOPs around 12–14 mmHg, the DEX-treatment paradigm raised the IOPs to 22–24 mmHg with fairly reproducible levels of OHT (8–10 mmHg increases in IOP) in several cohorts of animals during this almost year-long study of five compounds. Topical ocular drug instillation of the compounds in naïve mice that had only received a vehicle injection resulted in a 2-mmHg reduction in IOP with NS-304 and butaprost. However, a more robust drop in IOP (~4 mmHg) was observed in response to misoprostol, rivenprost, and PF-04217329 (Figure 3). In mice that had been subjected to DEX treatment, the compounds also induced a differential ocular hypotensive response with misoprostol, PF-04217329, and butaprost, causing the sharpest decrease and the largest overall reduction in IOP (~5–7 mmHg) as compared with the responses induced by both NS-304 and rivenprost (~3–4 mmHg) (Figure 3). The overall percentage IOP decreases from DEX-induced elevated IOP baselines induced by the various compounds in the DEX-induced OHT were as follows: misoprostol = 74.5%; PF-04217329 = 74.3%; butaprost = 65.2%, rivenprost = 58.4%, NS-304 = 55.3% (Table 1). In comparison, rivenprost (50% IOP reduction), latanoprost (IOP 50% reduction), and PF-04217329 (36% IOP reduction) exhibited a different rank order of potency and magnitude of IOP lowering in the mature DBA/2J mouse model of pigmentary glaucoma [64]. In a similar comparative manner, Table 3 shows the ability of several different classes of pharmacological agents to reduce IOP in normal mice.

## 3. Discussion

As recently reported by Maddineni et al. [20] and as observed in the current studies, weekly periocular injections of DEX cause an increased ECM deposition in the mouse TM with resultant reduced outflow facility and a sustained IOP elevation from baselines in multiple animals from 3-weeks onwards (Figure 2). When left untreated, the weeks-long DEX-induced OHT caused loss of RGCs and their axons with consequential retinal dysfunction as measured by electrophysiological methods [20].

The current studies revealed a differential onset of action, temporal profile, and magnitude of IOP-lowering induced by the five test PG compounds in normal (control) and DEX-treated mice. The IP- (NS-304) and EP4- (rivenprost) receptor agonists exhibited an overall shallower time course of action and a lower maximal ocular hypotensive response than the EP2- (PF-04217329; butaprost) and the mixed EP3/EP4/EP2- (misoprostol) receptor agonists (Figure 3). The fact that the profile of IOP-lowering activity of misoprostol resembled that of PF-04217329 and butaprost (Figure 3), the EP2 receptor agonist activity embedded in misoprostol (Table 1) was most likely contributing to the ocular hypotensive actions of misoprostol along with a contribution from the EP4 receptor. The latter conclusions stem from the previously reported data on the IOP-reducing actions of EP2 (ONO-AE1-259-01) and EP4 receptor (ONO-AE1-329) agonists in naïve mice, with EP1 (ONO-DI-004) and EP3 (ONO-AE-248) receptor agonists being inactive in mice [77]. A similar lack of activity of EP1- (17-phenyl-trinor PGE2) and EP3- (sulprostone) receptor activators in the monkey model of OHT was noted by Gabelt et al. [67]. In contrast, however, due to species variations, the EP3 receptor agonist, sulprostone (but not EP2, FP- or TP receptor agonists), robustly lowered IOP in rabbits [68].

The profile of the time-dependent reduction in IOP and the maximal effect induced by NS-304 and rivenprost were very similar (Figure 3). These represent novel data for IP- and EP4-selective compounds in normotensive and ocular hypertensive eyes of mice. The only other reports of an IP receptor agonist lowering IOP pertain to pigmented rabbits where iloprost, a stable prostacyclin analog, initially induced an increase in IOP followed by a lowering of IOP, and in glaucomatous Beagle dogs where only an ocular hypotensive response was observed [69]. Regarding EP4 receptor agonist activity, PF-04475270 [70] and 3,7-dithia-PGE2 [78] lowered IOP in OHT dogs and in OHT Cynomolgus monkeys by 30–50%, respectively. Thus, mice (normal and OHT with steroids), Beagle dogs, and OHT monkeys appear to respond similarly to topical ocular EP4 receptor agonists.

The EP2 receptor has been a target of many ocular researchers ever since Woodward et al. [79], Aihara [75], and Nilsson et al. [71] reported the ocular hypotensive activity of the EP2 receptor-selective agonists AH13205 (25–36% IOP decrease), AL-6598 (>50% IOP reduction) and butaprost (26–36% IOP decrease) in TM-lasered OHT monkey eyes, respectively. The free acid form of AL-6598 (AL-6556) possessed a high affinity and full agonist activity at the DP receptor and a partial agonist of EP2 receptor activity [72]. AL-6598 powerfully lowered IOP in Dutch-belt rabbits and OHT monkeys [72], and although AL-6598 induced significant hyperemia, it also lowered and controlled IOP in OHT/glaucoma patients [75]. Interestingly, the EP2-selective compounds butaprost and PF-0421729 were the most efficacious ocular hypotensive agents in the DEX-induced mouse model in the current studies, having a relatively fast onset of action compared to the other non-EP2 receptor compounds (NS-304; Rivenprost) (Figure 3; Table 2). More recently, a novel non-PG EP2 receptor-selective agonist, omidenepag isopropyl ester (OMDI), potently and efficaciously reduced IOP in rabbits, dogs, and lasered OHT Cynomolgus monkeys [73]. Importantly, OMDI potently and efficaciously lowered and controlled IOP as effectively as latanoprost in human subjects suffering from OHT, open-angle glaucoma (OAG), primary angle-closure glaucoma (PACG), and secondary glaucoma (see recent reviews [34,80,81]). Thus, it appears that EP2 receptor agonists can be useful in treating various forms of OHT induced by different mechanisms and under different experimental and clinical situations. In animal models of glaucoma/OHT, the ocular hypotensive effects of drug candidate molecules in the mouse, dog, and monkey seem to correlate fairly well, and at least for prostaglandins (FP–PG agonists), the steroid-induced rabbit-derived data also seem to correlate well with the other animals mentioned above (Table 2 and Table 3).

**Figure 4:**
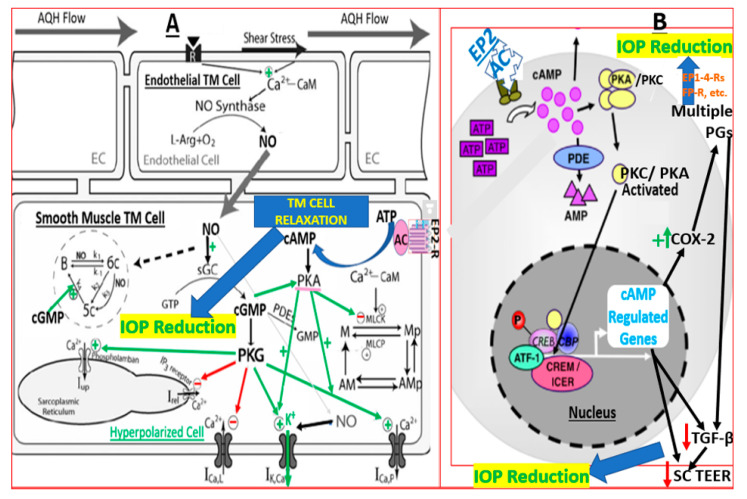
Schematic showing the interconnectivity and the signal transduction mechanisms and pathways within endothelial-like and smooth muscle-like TM cells in the TM tissue. The endothelial nitric oxide (NO) synthase (eNOS), NO, soluble guanylate cyclase, cGMP, PG EP2 receptors, and their signaling components all play a major role in the final IOP-reducing actions of EP2 receptor agonists (**A**). The cytoplasmic and nuclear components of the TM cells are also involved in terms of amplification of the overall biological response through upregulation of the gene that encodes cyclooxygenase-2, which generates endogenous PGs that cause IOP lowering via their cognate receptors (**B**). The heterogeneity of cells within the TM tissue (smooth muscle-like; endothelial-like, etc. as in (**A**)) permits cooperative local signaling to occur that ensures eventual homeostasis in terms of regulation of IOP.

In recent years, the collective knowledge about the potential mechanism(s) of action of the EP2 receptor-mediated IOP-lowering in OHT/glaucoma models has increased. A unified narrative about this can be presented as follows, depicted in the schematic in Figure 4A,B. Thus, EP2 receptor activation by the free acids of butaprost, PF-04217329, or PGE2 raises intracellular cAMP, which activates protein kinase A and induces efflux of K^+^ ions to hyperpolarize the TM cells, thereby relaxing the latter. This process is further enhanced via Ca^2+^-calmodulin and actin–myosin dephosphorylation, which also helps in the TM cell relaxation to stimulate the AQH outflow facility, thus reducing the IOP results (Figure 4A). Such TM cell relaxant activity may also be enhanced by NO released by endothelial cells within the TM, which generates cGMP by activating guanylate cyclase [82,83,84,85]. This cGMP activates protein kinase G to help reduce intracellular Ca^2+^, both by intracellular sequestration and through efflux via voltage-operated Ca^2+^ channels. All the aforementioned activities account for the early phase of IOP-lowering by EP2 receptor agonists (Figure 4A). The more durable ocular hypotensive effects of the latter compounds occur via nuclear activation of cAMP-regulated genes to upregulate cyclooxygenase-2 (COX-2) to produce and release a multitude of endogenous PGs, such as PGE2, PGF2a, and PGD2 which subsequently decrease IOP via their own preferred receptor and signal transduction systems [86,87]. Here, extensive TM remodeling by the MMPs released by EP2 receptors located in the TM and non-pigmented ciliary epithelial cells [88,89] and by FP receptors located in the ciliary muscle (CM) and TM cells [90,91] is responsible for lowering IOP (Figure 4B). Correlating with these factors and events are the observations that a year-long treatment of normotensive Cynomolgus monkey eyes with butaprost showed robust remodeling of the CM outflow pathway (also known as the uveoscleral (UVSC) outflow pathway), leading to increased UVSC outflow, which also lowers IOP [71]. Interestingly, the potent and EP2 receptor-selective agonist, omidenepag, causes IOP reduction by promoting AQH egress from both the conventional (trabecular) and UVSC pathways [92]. Finally, the EP2 receptor-coupled nuclear machinery can also down-regulate the generation/release of profibrotic cytokines (transforming growth factor-1/2 and connective tissue growth factor), thereby reducing collagen/fibronectin accumulation, and hence lowering and normalizing IOP [93].

Taken together, EP2 receptor agonists (butaprost and PF-04217329) and misoprostol most robustly reduced IOP in mice with DEX-induced OHT. IP and EP4 receptors (NS-304; Rivenprost) were less effective and exhibited a shallower onset and duration of action than the EP2 receptor agonists. Our future studies will endeavor to define the mechanism(s) of action of these compounds in this model of steroid-induced glaucoma and in other models of OHT. Furthermore, we will endeavor to determine the effect of IOP-lowering by these compounds on the fate of RGCs and their axons in future studies using the DEX-induced OHT mouse model.

## 4. Materials and Methods

### 4.1. Animals

Male C57BL/6J mice (Jackson Laboratories, Bar Harbor, ME, USA; age 2½–7 months) were kept in 12 h light/12 h dark conditions (lights on 0600 h) and fed with standard chow. All experimental procedures were conducted in accordance with and adherence to the ARVO Statement for the Use of Animals in Ophthalmic and Vision Research and the University of North Texas Health Science Center (UNTHSC) Institutional Animal Care and Use Committee Regulations and Guidelines, under the auspices of UNTHSC IACUC Protocols 2018-0036 and 2023-0027.

### 4.2. Ophthalmic Formulations

Prostanoid compounds used in the current studies (Figure 2) were purchased from Sigma-Aldrich (St. Louis, MO, USA, Product Number: PZ-0369 (PF-04217329)) or Cayman Chemical Company (Ann Arbor, MI, USA, Product Numbers: 13820 (Misoprostol), 13618 (Rivenprost), 10010411 (NS-304), and 13740 (Butaprost)).

### 4.3. Induction of Ocular Hypertension Using Dexamethasone (DEX)

For each compound to be tested, ten animals were selected and then divided into two cohorts of 5 animals. One cohort was given a once-weekly periocular injection of 20 μL (200 μg) of Dexamethasone Acetate (DEX) (10 mg/mL) in both eyes (OU). The other cohort received a once-weekly periocular injection of DEX-Vehicle. For periocular injections, animals were anesthetized in a chamber (isoflurane 2.5%, oxygen 0.8 L/min). Following the attainment of a surgical plane of anesthesia, animals were transferred to a mask. A 1” 33G needle attached to a 100 µL glass microsyringe (Hamilton Company, Reno, NV, USA) was inserted through the conjunctival fornix, and a 20 µL bolus of dexamethasone acetate (prepared 24 h previously, as described below) was injected into the retrobulbar area, immediately overlying Tenon’s capsule. Following injection, animals were placed in their cage and allowed to recover.

Full induction of OHT took place following four consecutive periocular injections of DEX.

### 4.4. Preparation of DEX for Periocular Injection

6–10 mg of Dexamethasone Acetate Anhydrous Micronized USP powder (Spectrum Chemical Manufacturing Corporation, Gardena, CA, USA & New Brunswick, NJ, USA DE122) was weighed on a balance and then transferred into a 2 mL tube containing two 5 mm diameter steel ball bearings. A suitable volume of vehicle was then transferred to the tube to make up a suspension of a final concentration of 10 mg/mL. The suspension was then ball-milled using a bench-top vortex mixer for 5 min. Following that, the suspension was wrapped in aluminum foil and placed on a rotating mixer overnight for injection the following day. A separate tube filled only with the vehicle was prepared simultaneously in an identical manner. The DEX suspension and vehicle solutions were vortex-mixed once more for 30 s immediately prior to injection.

The vehicle was prepared by Perrone Compounding Pharmacy, Fort Worth, TX, USA, and consisted of the following: Sodium chloride USP (granular), creatinine NF powder, edetate disodium USP dihydrate powder, carboxymethylcellulose sodium USP (medium viscosity), polysorbate 80 NF liquid, benzyl alcohol NF liquid, sodium bisulfite powder, and sterile water (PF).

### 4.5. IOP Measurement/Direct Ophthalmoscopy

IOP was determined once-weekly in restrained behaviorally trained conscious animals using a TonoLab^®^ rebound tonometer (Colonial Medical Supply, Franconia, NH, USA), following our previously published methodology [94]. At the time of IOP measurement, both eyes of restrained animals were also examined by direct ophthalmoscopy (Specialist Model Ophthalmoscope, Keeler Instruments, Windsor, UK). None of the eyes examined showed any signs of discharge, corneal edema, corneal opacity, iridial hyperemia, conjunctival hyperemia, or lenticular cataract, nor any other ocular pathology at any point throughout the study.

Behavioral training for IOP measurement was conducted in 3 sessions prior to the commencement of any actual experiment. During the session, animals were placed inside the wide end of a Mouse DecapiCone (Model MDC-200, Braintree Scientific Inc., Braintree, MA, USA), which had been previously trimmed at its narrow end such that the animal’s face could comfortably protrude but not its ears. The animal thusly secured in the DecapiCone was then in turn secured in a Mouse Restraint device (Part MR01C, Colonial Medical Supply Co., Inc., Londonderry, NH, USA) using the Velcro straps provided with the restrainer. Care was taken not to set the straps tightly around the thorax of the animal. Secured in this way, the animal’s eyes were clearly accessible in an unobstructed manner, but the animal was not able to move its head from side to side or up and down. The restrainer with the animal was then placed on top of the flat platform of an adjustable laboratory jack. The height of the jack was adjusted until the left eye was lined up with the probe of a Tonolab Impact Tonometer (clamped securely in an upright stand) at a distance of 2–3 mm from the cornea. Six complete IOP readings were taken over the course of approximately one minute. The animal was then turned so that the right eye was now lined up with the tonometer probe, and six more complete IOP measurements were taken. Following these measurements, the animal was released from the restrainer and DecapiCone and returned to its cage. The entire time between an animal first entering the DecapiCone and being returned to its cage was less than 3 min.

During the second and third training session, all procedures were identical except that immediately following IOP measurement, both eyes were also given a very brief and cursory ophthalmoscopic examination, in order to acclimatize the animals to the brief bright light associated with this procedure.

Any animal that would not settle and remain calm during the third training session or that would not yield consistent IOP readings was not used in further studies.

Actual experimental IOP readings and ophthalmoscopic scores were obtained in a manner similar to the behavioral training sessions, and over a similar time-frame, of less than 3 min per animal.

### 4.6. Topical Ocular Dosing

Drugs were diluted to 1 mg/mL concentration using DMSO (Sigma Chemical Company, St. Louis, MO, USA) or methyl acetate (Sigma Chemical Company, St. Louis, MO, USA) as appropriate. The topical ocular vehicle was made up by dissolving 1 mL of DMSO or methyl acetate in 4 mL of sterile PBS. When dosing with prostanoid compounds, the indicated prostanoid formulation (or prostanoid formulation vehicle) was administered topical ocular as a single 5 μL drop (incorporating 5 μg of drug) to both eyes of each animal using a 10 μL adjustable pipette (Eppendorf, Enfield, CT, USA) twice per day, every day (including weekends) at 0900 h and 1700 h. DMSO or methyl acetate were the experimental dissolution liquids utilized in current studies, and these are not intended or recommended for future medical veterinary or human medicinal uses. Likewise, the dose of the compounds used in the current comparative studies was selected on the basis of many literature reports where the same or similar classes of prostanoid/prostaglandin compounds were utilized (e.g., Table 2; Refs. [20,30,37,38,61,64,65,66,67,68,69,70,71,72,73,74,75]). Consideration was also given to the relative PG receptor selectivity of each of the compounds tested (Table 1).

### 4.7. Statistical Analysis

Following tests of normality (Shapiro-Wilk) and equality of variance (Browne-Forsythe), a 2-factor analysis of variance (ANOVA) was utilized, followed by Holm-Sidak post-hoc testing (all pairwise multiple comparisons) to compare IOP in drug-treated versus drug-Veh treated eyes, in each drug treatment group. All statistical tests were performed using the software provided by SigmaPlot 15.0 *p* values of <0.05 were considered significant. All data are presented as Mean ± SDM or Mean ± SEM, as indicated.

## 5. Conclusions

The current studies have revealed apparent differences in the time course and maximal IOP reduction profiles of four prostaglandin receptor-selective drugs in the mouse steroid-induced OHT glaucoma model. The relative receptor expression levels, the residence time of the compounds at the receptors, the effectiveness of signal transduction associated with EP2, EP3, EP4, and IP receptors in the target tissues, and perhaps differential metabolism of the compounds most likely account for the differences observed. However, all these drugs are known to induce or modulate the production of cAMP within target cells [16,34,37,38,64,70,73,78,79,89] and appear to recruit multiple intracellular and perhaps nuclear signaling mechanisms (Figure 4), which may explain their high efficacy relative to other drugs such as FP receptor agonists (e.g., latanoprost [58,61]), and other receptor agonists [Table 2] in this model of OHT. Also, since enzyme inhibitors such as dorzolamide, Y39983 and netarsudil, beta-blockers (e.g., timolol), and intracellular Ca^2+^-mobilizing agents (e.g., pilocarpine, latanoprost, and losartan) appeared not to be as potent and effective as the cAMP-inducing drugs tested in reducing IOP in the steroid-induced animal models (Table 2), the data suggest that future treatments of OHT triggered by corticosteroids may respond best to cAMP-generating drugs. However, this conclusion needs to be confirmed in additional studies. Likewise, the use of a more clinically acceptable solvent other than DMSO, the conduction of dose–response studies for each of the tested drugs, and further confirmation of the drug effects in other species would add value to the current observations made in the mice.

## Figures and Tables

**Table 1:** Human prostanoid receptor type and sub-type receptor binding inhibition constants of the active moiety free acid forms of various test compounds.

	Major Prostanoid Receptor Types and Some Sub-Types							
	DP1	EP1	EP2	EP3	EP4	FP	IP	TP
	Receptor Binding Inhibition Constants (K_i_, nM)							
Latanoprost free acid	54,567	1750	39,667	6503	100,000	**3**	100,000	16,300
Butaprost free acid	12,097	27,721	**91**	1643	19,104	100,000	54,836	19,667
PF-04217329 free acid		3200	**15**	3200	3200			
Misoprostol free acid	15,876	11,935	**34**	**8**	**23**	9382	100,000	53,047
Rivenprost free acid		10,000	620	56	**1**			
NS-304 free acid (MRE-269)		>10,000	5800	>10,000	4900	>10,000	**20**	>10,000

Receptor radioligand binding competition data are from Abramovitz et al. [36] for most of the compounds generated against human-cloned receptors expressed in a cell line that was previously devoid of the receptor under study. Data for PF-04217329 and NS-304 are from manuscripts by Prasanna et al. [37] and Asaki et al. [38], respectively. Note that the receptor affinity of the compound is inversely related to the K_i_ value. Thus, the lower the K_i_ value, the higher the affinity. The blue bolded colored numbers reflect the highest affinity of the compound for that preferred receptor type.

**Table 3:** Ocular hypotensive activity of various compounds in naïve normotensive adult mice.

Compound	Receptor/Enzyme Target	Maximum IOP-Lowering Observed (% From Baseline)	Reference
Tafluprost	FP–PGR agonist	24%	[65]
Latanoprost	FP–PGR agonist	20%	
Travoprost	FP–PGR agonist	21%	
Unoprostone	FP–PGR agonist	11%	
Latanoprost	FP–PGR agonist	21% (16%)	[74]
Travoprost	FP–PGR agonist	25% (19%)	
Bimatoprost	FP–PGR agonist	20% (13%)	
Unoprostone	FP–PGR agonist	14% (11%)	
Tafluprost	FP–PGR agonist	22%	[66]
Latanoprost	FP–PGR agonist	22%	
Bunazosin	α-1R antagonist	22%	
Dipivefrin	α-2R agonist	27%	
Timolol	β-blocker	17%	
Dorzolamide	Carbonic anhydrase inhibitor	11%	
Pilocarpine	Muscarinic receptor agonist	11%	
Betaxolol	β-blocker	38%	[76]
Latanoprost	FP–PGR agonist	34%	
Brimonidine	α-2R agonist	16%	

## Data Availability

The data will be made available upon request.
